# Calnexin Regulates Apoptosis Induced by Inositol Starvation in Fission Yeast

**DOI:** 10.1371/journal.pone.0006244

**Published:** 2009-07-16

**Authors:** Renée Guérin, Pascale B. Beauregard, Alexandre Leroux, Luis A. Rokeach

**Affiliations:** Department of Biochemistry, Université de Montréal, Montréal, Québec, Canada; University of Texas MD Anderson Cancer Center, United States of America

## Abstract

Inositol is a precursor of numerous phospholipids and signalling molecules essential for the cell. *Schizosaccharomyces pombe* is naturally auxotroph for inositol as its genome does not have a homologue of the *INO1* gene encoding inositol-1-phosphate synthase, the enzyme responsible for inositol biosynthesis. In this work, we demonstrate that inositol starvation in *S. pombe* causes cell death with apoptotic features. This apoptotic death is dependent on the metacaspase Pca1p and is affected by the UPR transducer Ire1p. Previously, we demonstrated that calnexin is involved in apoptosis induced by ER stress. Here, we show that cells expressing a lumenal version of calnexin exhibit a 2-fold increase in the levels of apoptosis provoked by inositol starvation. This increase is reversed by co-expression of a calnexin mutant spanning the transmembrane domain and C-terminal cytosolic tail. Coherently, calnexin is physiologically cleaved at the end of its lumenal domain, under normal growth conditions when cells approach stationary phase. This cleavage suggests that the two naturally produced calnexin fragments are needed to continue growth into stationary phase and to prevent cell death. Collectively, our observations indicate that calnexin takes part in at least two apoptotic pathways in *S. pombe*, and suggest that the cleavage of calnexin has regulatory roles in apoptotic processes involving calnexin.

## Introduction

The endoplasmic reticulum (ER) is a central organelle playing crucial roles in several cellular processes. The ER is at the center of the synthesis and the folding of secreted, membrane-bound and some organelle-targeted proteins [1], [2]. To assist in the protein folding process, the ER contains molecular chaperones, several co-factors such as ATP and Ca^2+^, and an optimal oxidizing environment to facilitate disulphide-bond formation [1], [2], [3]. In addition to its protein folding roles in the secretory pathway, the ER is crucial for other fundamental cellular processes including lipid biosynthesis, membrane biogenesis, and Ca^2+^ storage [4].

Perturbations in the ER homeostasis by stresses such as lipid and glycolipid imbalances, alterations in the levels of Ca^2+^, or modification in redox state, negatively affect the protein folding capacity of this organelle [5], [6], [7]. These adverse conditions, referred to as ER stress, result in the accumulation and aggregation of unfolded or incompletely folded proteins. The functions of the ER are tightly regulated. To counteract ER stress and restore its full protein-folding capacity, the ER responds by inducing a stress-response pathway called UPR, for Unfolded Protein Response [8], [9]. The UPR mechanism is well conserved from yeast to mammals. The UPR stops general protein synthesis and stimulates the transcription of genes coding for ER-folding factors such as molecular chaperones and foldases [5], [6], [7], [10], [11]. Concomitantly, the cell improves the ERAD (ER-Associated Degradation) pathway in order to degrade the unfolded proteins present is the ER [12], [13], [14]. These actions allow the ER to stabilize its environment and ensure cell survival. A major regulator of the UPR is the ER transmembrane protein Ire1p (Inositol requiring kinase I) which is conserved from fungi to mammals [8], [9]. Ire1p is an endoribonuclease that is activated by homodimerization and autophosphorylation, and whose downstream effect is to stimulate the transcription of genes encoding ER chaperones and other factors involved in all stages of the secretory pathway. So far, Ire1p is the only factor identified in yeast to transduce the UPR, but mammalian cells contain additional transducers called PERK and ATF6 [9], [15]. If this initial suite of actions is not able to restore ER homeostasis, the UPR switches its downstream effects from pro-survival to pro-death. Prolonged ER stress induces via IRE1, PERK and ATF6 an apoptotic pathway involving the Bcl-2 family of proteins, which act at the level of the mitochondria and the ER [6], [7], [16].

Apoptosis is a central molecular process first identified in multicellular organisms for its crucial roles in development and in regulation of many diseases. Apoptosis is a tightly regulated form of programmed cell death that is characterized by specific biochemical and morphological features such as cell rounding and shrinkage, chromatin breakage, nuclear fragmentation and activation of caspases [17], [18]. Mounting evidence accumulated in the last ten years established that unicellular organisms such as yeasts undergo apoptotic cell death [19], [20], [21]. Apoptosis in yeast is induced by numerous conditions including DNA damage, aging, replication defects, deficiency in triacylglycerols, ER stress, and mating [19], [22]. The yeast genomes encode several homologues of proteins characterized for their involvement in apoptosis including Yca1/Pca1, AIF, EndoG, HtrA2/Omi and IAP [23], [24], [25], [26], [27], [28]. As is for mammalian cells, the involvement of these factors and their interaction in different apoptotic pathways are under current investigation.

Inositol is a precursor for numerous molecules including inositol-containing phospholipids, inositol esters and phosphorylated versions of inositol playing central roles in membrane integrity, cell signalling and vesicular traffic [29], [30], [31]. The pathway involving inositol 1,4,5-triphosphate (IP_3_) has been extensively characterized. IP_3_ derives from the hydrolysis of phosphatidylinositol 4,5-biphosphate (PIP_2_) by phospholipase C (PLC), thereby producing IP_3_ and DAG (diacylglycerol). IP_3_ acts a second messenger via its binding to the IP_3_R receptor on the ER membrane [32]. The binding of IP_3_ to its receptor provokes the release of the ER-stored calcium into the cytoplasm, which in turn elicits a range of cellular responses [33]. Calcium release from vacuoles following IP_3_-signalling was also observed in *Saccharomyces cerevisiae* [34]. Inositol can be synthesized by intracellular processes involving conversion of glucose-6-phosphate (G6P) into inositol monophosphate (IP_1_) through a set of complex reactions of oxidation/reduction which are mediated by a unique enzyme, the inositol-1-phosphate synthase (*INO1*) [35], [36]. The *INO1* gene was identified in various species of yeasts, protozoa, plants and mammals [37], [38], [39]. Deletion of *INO1* and mutation in other genes such as *IRE1* cause inositol auxotrophy in *S. cerevisiae* [40], [41]. In this yeast, expression of *INO1* is under the control of *IRE1* via *HAC1*, two key players of the UPR pathway [42], [43].

*Schizosaccharomyces pombe* is considered a good model to study the involvement of inositol in cell pathways because this yeast is naturally auxotroph for inositol due to the absence of a gene coding for an inositol-1-phosphate synthase [44], [45]. Studies in *S. pombe* have shown that absence of inositol in the culture medium is lethal for this yeast, and that partial depletion provokes sexual sterility with no effects on growth [46], [47], [48], [49], [50], [51]. Although *S. pombe* cells die in the absence of inositol, they are able to survive longer than *S. cerevisiae* cells auxotroph for inositol as result of genetic manipulation (*Δino1* cells) [49].

Calnexin is an ER transmembrane chaperone playing key roles in translocation, in protein folding, and in the quality control of newly synthesized polypeptides [1], [2], [52]. Structurally, calnexin is a type I transmembrane protein of the ER containing a large lumenal domain, a transmembrane domain (TM), and a short cytosolic tail. The lumenal domain folds into a globular structure formed by the C- and N-terminal extremities, and a hairpin structure formed by the highly conserved central domain (hcd), which is the most conserved calnexin domain across species. Calnexin interacts with client proteins via glycan-lectin or protein-protein interactions [52], [53], [54], [55], [56], [57], [58], [59], [60], [61].

The knockout of calnexin in mice causes early postnatal death and severe motor disorders and is lethal in *S. pombe*, thus pointing to the critical cellular roles of this protein [62], [63], [64]. We showed that certain calnexin chaperone-deficient mutants are viable. Interestingly, this demonstrates that the essentiality of calnexin is not its chaperone activity but another yet to be defined cellular role [58], [65], [66].

Several studies published in the recent years indicate that calnexin is involved in apoptotic processes induced by ER stresses. First indications came from a report showing that the cytosolic tail of *S. pombe* calnexin is required for cell death mediated by the heterologous expression of mammalian Bak, suggesting that calnexin can form a complex with lethal partners in apoptotic situations [67]. In mammalian cells, it was shown that calnexin-deficient cells are more resistant to apoptosis [68], [69]. It was suggested that calnexin could act as a scaffold for the cleavage of the ER transmembrane apoptotic protein Bap31 by caspase 8 in ER-stress conditions [70]. In addition, calnexin in mammalian cells was shown to be sensitive to caspase cleavage under stress conditions [71]. It was proposed that this cleavage could have a role in the transduction of an apoptotic signal. More recently, we showed that overexpression of calnexin in *S. pombe* causes apoptosis and that this induction requires the anchoring of calnexin to the ER membrane [72]. We also demonstrated that apoptosis induced by tunicamycin is less efficient in cells containing only a lumenal version of calnexin, thus pointing to the importance of both the membrane anchoring of calnexin and of its cytosolic tail.

Here, we demonstrate that inositol starvation induces cell death in *S. pombe* via an apoptotic pathway dependent on the metacaspase Pca1p and that is modulated by the UPR transducer Ire1p. We show that calnexin is cleaved under normal conditions when cells approach stationary phase. While a lumenal version of calnexin exacerbates the apoptosis provoked by inositol starvation, co-expression of a mutant spanning the TM and C-terminal tail significantly reduces the levels of apoptosis. Our work suggests that the TM and C-terminal tail of calnexin are involved in apoptotic signalling when inositol is depleted.

## Results

### Inositol starvation induces apoptotic cell death in *S. pombe*

**Figure 1:**
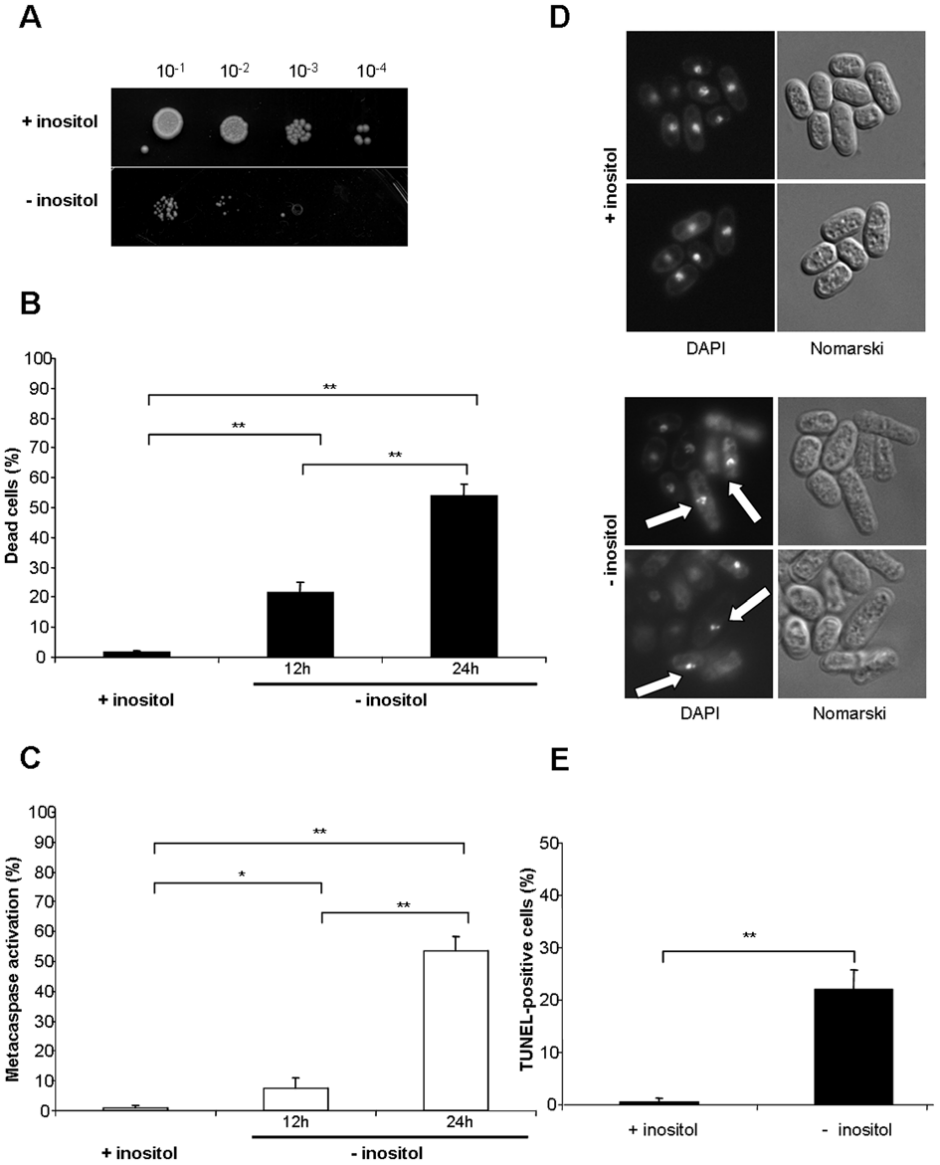
Inositol starvation induces apoptotic cell death. (A) Survival of cells cultured for 48 h in MM with or without inositol was assayed by serial dilution on media containing inositol. Samples of 10 µl of four 10-fold serial dilutions (10^−1^–10^−4^) of cells at OD_595_ 1 were spotted on selective MM with inositol, and incubated at 30°C for 7 days (see *Materials and Methods*). (B) Percentage of dead cells measured by staining with the fluorescent vital dye Phloxin B. Cells cultured for 24 h in MM containing inositol or for 12 h and 24 h in MM without inositol, were stained with Phloxin B and fluorescent cells were quantified by FACS. Stained cells were considered as dead. (C) Metacaspase activation. The fluorescent probe FITC-VAD-FMK was used to measure metacaspase activation by FACS after 12 and 24 h of growth in MM without inositol, or after 24 h of growth in MM containing inositol, as described in *Materials and Methods*. (D) Nuclear fragmentation. Cells cultured for 48 h in MM with or without inositol were stained with DAPI and visualized under fluorescence microscopy; Nomarski fields are shown. White arrows indicate fragmented nuclei. (E) DNA breakage. TUNEL assay was carried out with cells cultured for 48 h in MM with or without inositol. The percentage of stained cells was measured by FACS. The significance of differences in the results was evaluated by a Student's *t* test pairwise calculated between the different conditions assayed. **p<0.01 and *p<0.05.

Inositol starvation induces cell death in fission yeast [44], [45]. This effect is due to the inability of *S. pombe* to synthesize inositol because of the lack of a homologue the *INO1* gene in its genome. Inositol starvation in *S. cerevisiae* also provokes cell death when *INO1* is defective, indicating a crucial role of inositol in the physiology of the cell. To characterize the death resulting from inositol depletion in *S. pombe*, we first measured the capacity of cells to grow on media containing inositol after 48 hours of culture in media without inositol. As expected, cells cultured in media deprived of inositol show a dramatic reduction in the ability to form colonies compared to cells cultured in media containing inositol (Figure 1A). The death phenotype was confirmed by staining with the fluorescent vital dye Phloxin B, followed by quantification by fluorescence-activated cell sorting (FACS). As shown in Figure 1B, about 25% of cells are stained after 12 hours of inositol starvation and more than 50% after 24 hours, as compared to near 0% for the unstarved cells. The cell death observed following inositol starvation is specific since starvation in adenine or leucine for 12 h and 24 h do not promote cell death (Supplemental Figure S1). Next, to determine the type of cell death we measured apoptotic markers in cells starved for inositol. As we have previously demonstrated, *S. pombe* cells undergoing apoptotic death show specific phenotypes including metacaspase activation, DNA breakage and nuclear fragmentation [72]. Using the fluorescent probe FITC-VAD-fmk in FACS analyses, cells submitted to inositol starvation for 12 and 24 hours show respectively 15 and 50% of fluorescence, compared to near 0% for cells that were not starved (Figure 1C). Fluorescence microscopy with DAPI revealed nuclear fragmentation for cells cultured for 48 h without inositol (Figure 1D). By contrast, cells cultured in inositol-containing medium exhibited round and intact nuclei. Consistent with the DAPI staining results, a TUNEL assay shows that only cells starved for inositol display DNA breakage (Figure 1E). Taken together, these results confirm that depletion of inositol is lethal for *S. pombe* and indicate that this death is mediated via an apoptotic mechanism.

### The metacaspase Pca1p is required to mediate apoptosis induced by inositol starvation

**Figure 2:**
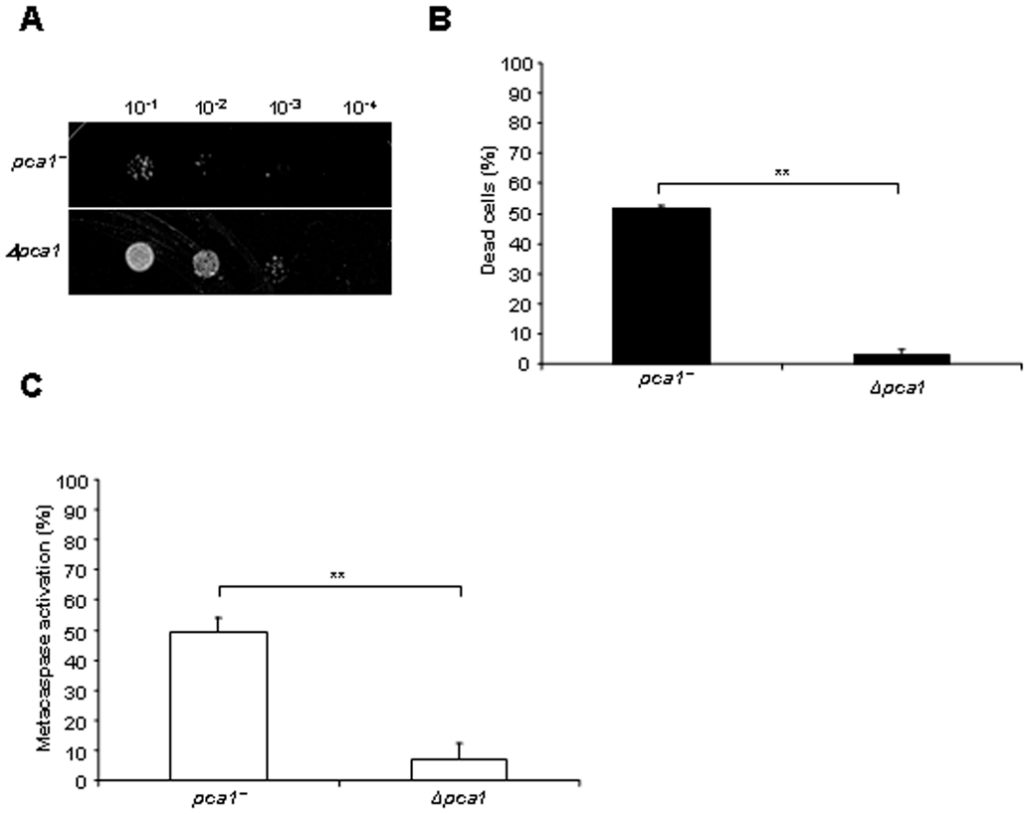
The metacaspase Pca1p is required for apoptosis induced by inositol starvation. (A) Survival of *pca1^+^* and *Δpca1* cells cultured for 48 h in MM without inositol was assayed by serial dilution on solid media containing inositol. Samples of 10 µl of four 10-fold serial dilutions (10^−1^–10^−4^) of cells at OD_595_ 1 were spotted on selective MM plates with inositol, and incubated at 30°C for 7 days (see *Materials and Methods*). (B) Percent of dead cells measured by staining with the fluorescent vital dye Phloxin B. *Pca1^+^* and *Δpca1* cells grown for 24 h in MM without inositol were stained with Phloxin B, and fluorescent cells were quantified by FACS. Stained cells were considered as dead. (C) Metacaspase activation. The fluorescent probe FITC-VAD-FMK was used to measure metacaspase activation by FACS of *pca1^+^* and *Δpca1* cells after 24 h of culture in MM without inositol, as described in *Materials and Methods*. The significance of differences in the results was evaluated by a Student's *t* test pairwise calculated between the different conditions assayed. **p<0.01 and *p<0.05.

The *S. pombe* genome encodes several characterized homologues of factors associated with apoptotic pathways in mammalian cells [23], [24], [25], [26], [27]. In *S pombe*, Pca1p is the only caspase-like protein identified so far, and the most studied factor for dependence in apoptosis [73], [74]. As this is the first time that death by inositol starvation is described as apoptotic in fission yeast, we investigated whether this process requires the metacaspase Pca1p. To this end, *Δpca1* cells were cultured in inositol-less medium for 48 h and spotted on inositol-containing plates. A dramatic diminution in the death level induced by inositol starvation was observed in a *Δpca1* strain compared to the wild-type control (Figure 2A). The reduction in the levels of cell death in the absence of *pca1^+^* was confirmed by Phloxin B staining. Following 24 h of inositol starvation, the *Δpca1* strain exhibited practically no Phloxin B staining compared to more than 50% for the wild-type control (Figure 2B). Metacaspase activation was measured with the fluorescent probe FITC-VAD-FMK. Congruently, a significant reduction in the level of metacaspase activation was observed in a *Δpca1* background compared to the wild-type control (Figure 2C). These results demonstrate the importance of the metacaspase Pca1p in the apoptotic pathway induced by the absence of inositol.

### The UPR transducer Ire1p affects the apoptosis induced by inositol starvation

**Figure 3:**
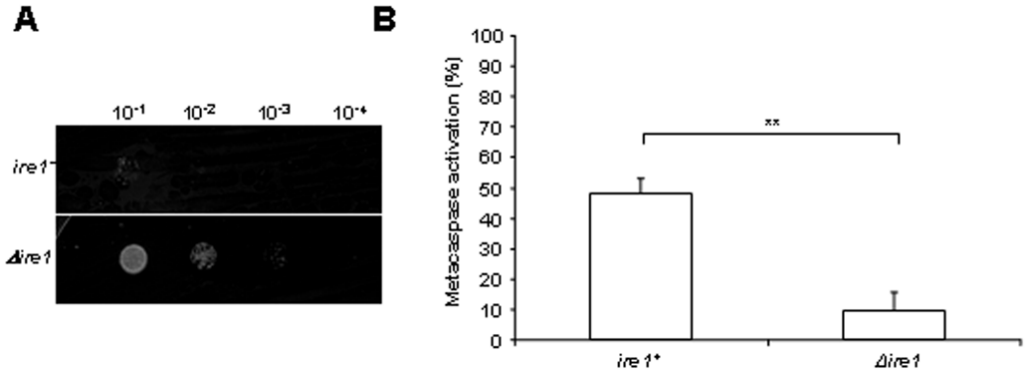
The UPR transducer Ire1p affects the apoptosis induced by inositol starvation. (A) Survival of *ire1^+^* and *Δire1* cells cultured for 48 h in MM without inositol was assayed by serial dilution on solid media containing inositol. Samples of 10 µl of four 10-fold serial dilutions (10^−1^–10^−4^) of cells at OD_595_ 1 were spotted on selective MM plates with inositol, and incubated at 30°C for 7 days (see *Materials and Methods*). (B) Metacaspase activation. The fluorescent probe FITC-VAD-FMK was used to measure metacaspase activation by FACS of *ire1^+^* and *Δire1* cells after 24 h of culture in MM without inositol, as described in *Materials and Methods*. The significance of differences in the results was evaluated by a Student's *t* test pairwise calculated between the different conditions assayed. **p<0.01 and *p<0.05.

Inositol is essential for the survival of *S. pombe* because its genome does not have an *INO1* homologue encoding for the inositol-1-phosphate synthase enzyme responsible for the biosynthesis of inositol from glucose-6-phosphate [35], [36], [44], [45]. In *S. cerevisiae*, *INO1* is under the control of the UPR via *IRE1* and *HAC1*, and the knockout of *IRE1* leads to inositol auxotrophy [42], [43]. To investigate if *ire1^+^* is involved in apoptosis induced by inositol starvation in *S. pombe*, *Δire1* cells were cultured in the absence of inositol for 48 h and the death levels were measured by spotting on inositol containing plates. A significant improvement in the levels of survival was observed for the *Δire1* strain in comparison to the wild-type control (Figure 3A). The induction of metacaspase was measured by FITC-VAD-FMK staining and FACS analysis. Here again, a dramatic reduction in the levels of metacaspase activation was observed for the *Δire1* strain compared to wild-type cells. The levels of metacaspase activation dropped to about 15% in the absence of Ire1p compared to near 50% in its presence (Figure 3B). These results demonstrate that Ire1p significantly influences the apoptotic cell death induced by inositol starvation in *S. pombe*, albeit not totally as a certain level of death is measured in *Δire1* cells.

### A lumenal version of calnexin is more sensitive to apoptosis induced by inositol starvation

**Figure 4:**
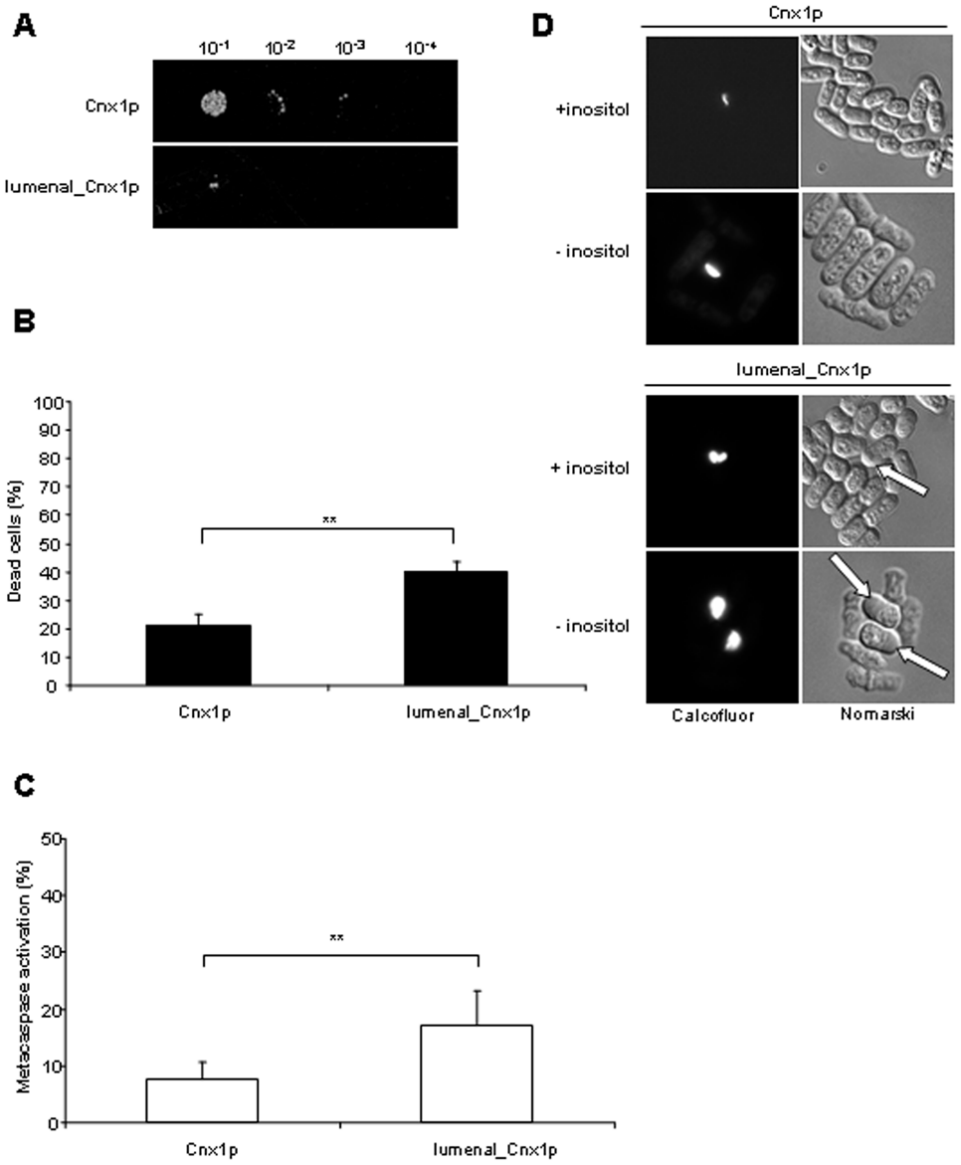
Increased apoptosis induced by inositol starvation in cells expressing a lumenal calnexin version. (A) Survival of Cnx1p and lumenal_Cnx1p strains cells cultured for 48 h in MM depleted of inositol was assayed by serial dilution on media containing inositol. Samples of 10 µl of four 10-fold serial dilutions (10^−1^–10^−4^) of cells at OD_595_ 1 were spotted on selective MM plates containing inositol, and incubated at 30°C for 7 days (see *Materials and Methods*). (B) Percent of dead cells measured by staining with the fluorescent vital dye Phloxin B. *cnx1^+^* and *lumenal_cnx1* strains cultured for 12 h in MM depleted in inositol were stained with Phloxin B and the fluorescent cells were quantified by FACS. Stained cells were considered as dead. (C) Metacaspase activation. The fluorescent probe FITC-VAD-FMK was used to measure metacaspase activation by FACS of *cnx1^+^* and *lumenal_cnx1* strains after 12 h of growth in MM without inositol, as described in *Materials and Methods*. (D) Cell morphology. Cells expressing only Cnx1p or lumenal_Cnx1p were cultured for 48 h in MM without inositol and stained with Calcofluor-white, and visualized under fluorescence microscopy; Nomarski fields are shown. White arrows indicate the vesicles containing Calcofluor-stainable material accumulating in the *lumenal_cnx1* strain. The significance of differences in the results was evaluated by a Student's *t* test pairwise calculated between the different conditions assayed. **p<0.01 and *p<0.05.

We have previously demonstrated that calnexin is involved in apoptosis caused by ER stress in *S. pombe* [72]. To examine if calnexin is part of the death pathway induced by the absence of inositol, we compared WT cells to a strain expressing the lumenal_Cnx1p mutant which lacks the trasmembrane domain (TM) and the cytosolic tail of this ER protein. As shown in Figure 4B, in inositol starvation the cells expressing the lumenal_Cnx1p mutant as the only version of calnexin exhibited a dramatic increase in the death levels, as measured by the ability to form colonies (Figure 4A). Moreover, the *lumenal_cnx1* cells showed a close to 2-fold increase in cell death measured by Phloxin B staining after 12 h of starvation (Figure 4B). Calcofluor-white is a fluorescent dye that stains a cell-wall polysaccharide, probably chitin, accumulating in the septa of *S. pombe* [65], [75]. Interestingly, inositol-starved *lumenal_cnx1* cells accumulate large, round vesicles containing material that is highly stained with Calcofluor (Figure 4D). These vesicles were also observed when *lumenal_cnx1* cells were cultured in inositol-containing media but at a much lower frequency, as compared to deprivation conditions (Figure 4D). This phenotype exacerbated by inositol starvation is also observed when calnexin mutants are submitted to heat stress in inositol-containing medium, conditions in which they exhibit cell-wall defects [65]. Thus, this Calcofluor-staining phenotype suggests a link between inositol and cell-wall biosynthesis.

In agreement with the levels of cell death observed by Phloxin B, cells expressing only the lumenal version of calnexin exhibited a close to 2-fold increase in metacaspase activation following 12 h of growth in media without inositol as compared to the c*nx1^+^* strain (Figure 4C). These results implicate calnexin in the death cascade triggered by inositol starvation, and point to a role of its TM and cytosolic tail in this apoptotic pathway.

### Co-expression of the cytosolic tail and TM with lumenal_Cnx1p reduces the sensitivity to inositol starvation

**Figure 5:**
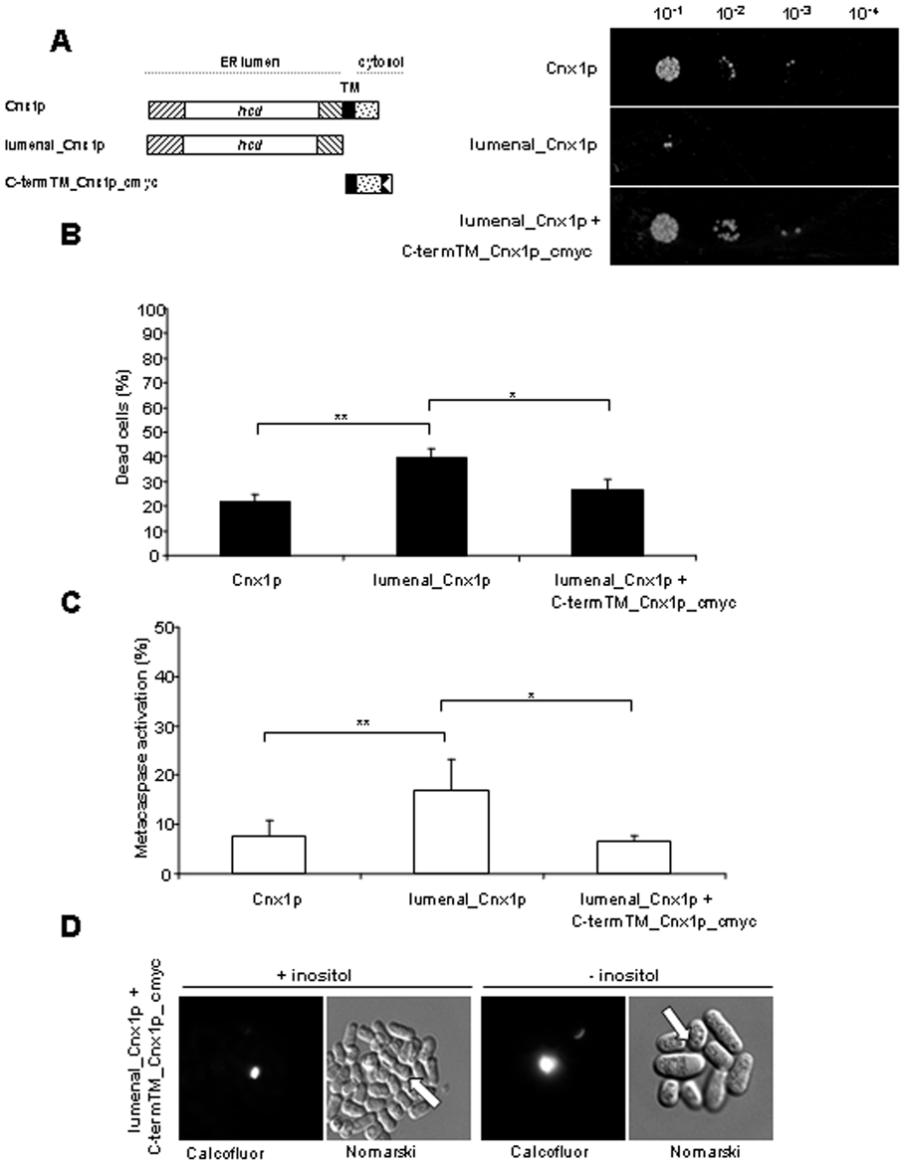
C-terminalTM_Cnx1pcmyc reverses the augmentation of apoptosis induced of the *lumenal_cnx1* strain caused by inositol starvation. (A) Schematic representation of WT calnexin and of calnexin mutants used in this study. The mutant lumenal_Cnx1p spans the lumenal domain of calnexin (415 aa) and is truncated of the transmembrane domain (TM) and the cytosolic tail. The mutant C-termTM_Cnx1p_cmyc spans the TM domain (23 aa) and the cytosolic tail (48 aa) fused to the *cmyc* tag. (B) Percent of dead cells measured by staining with the fluorescent vital dye Phloxin B. *cnx1^+^*, *lumenal_cnx1* and *lumenal_cnx1*+*C-termTM_cnx1_cmyc* strains cultured for 12 h in MM without inositol were stained with Phloxin B, and fluorescent cells were quantified by FACS. Stained cells were considered as dead. (C) Metacaspase activation. The fluorescent probe FITC-VAD-FMK was used to measure metacaspase activation by FACS of *cnx1^+^*, *lumenal_cnx1* and *lumenal_cnx1*+*C-termTM_cnx1_cmyc* strains after 12 h of cultured in MM depleted of inositol, as described in *Material and Methods*. (D) Cell morphology. Cells expressing only Cnx1p, lumenal_Cnx1p or lumenal_Cnx1p+C-termTM_Cnx1p_cmyc were cultured for 48 h in MM without inositol and stained with Calcofluor-White, and visualized under fluorescence microscopy; Nomarski fields are shown. White arrows indicate the accumulation of Calcofluor-stained material in the *lumenal_cnx1* strain. The significance of differences in the results was evaluated by a Student's *t* test pairwise calculated between the different conditions assayed. **p<0.01 and *p<0.05.

Since lumenal_Cnx1p is a calnexin mutant with full chaperone activity [58], we hypothesized that the increased effect on apoptosis induced by inositol starvation could be due to the absence of the cytosolic tail and the TM. To test this hypothesis, we co-expressed lumenal_Cnx1p with the mutant C-termTM_Cnx1p_cmyc, which spans the TM and cytosolic tail of calnexin (Figure 5A). Expression of the C-termTM_Cnx1p_cmyc mutant in conjunction with the lumenal_Cnx1p reduced the cell death to wild-type levels, as measured by Phloxin B staining (Figure 5B). The same reduction was observed for the levels of metacaspase activation when lumenal_Cnx1p was co-expressed with the C-termTM_Cnx1p_cmyc mutant (Figure 5C). This particular effect is not due to variations of *lumenal_cnx1* expression because the level of luminal_Cnx1p remains unchanged in the presence of a plasmid expressing the C-termTM_Cnx1p mutant (Supplemental Figure S2). Interestingly, the Calcofluor-staining phenotype observed for the *lumenal_cnx1* cells submitted to inositol starvation was not completely reverted in the presence of the C-termTM_Cnx1p_cmyc mutant (Figure 5D). The levels of large round structures stained with Calcofluor in media with or without inositol is the same whether the mutant C-termTM_Cnx1p_cmyc is co-expressed or not. These observations indicate that the anchoring of the C-terminal tail of calnexin to the ER membrane is important in the response to the apoptotic signal induced by inositol starvation. However, the cell-wall defects observed by Calcofluor staining appear to be more attributable to the requirement for an intact, i.e. wild-type calnexin. The importance of calnexin in cell-wall integrity was already shown in *S. cerevisiae* [76], and in *S. pombe* as we observed previously the same defect in calnexin mutants at 37°C [65], [66], [77].

### Calnexin is cleaved within the lumenal domain under normal culture conditions

**Figure 6:**
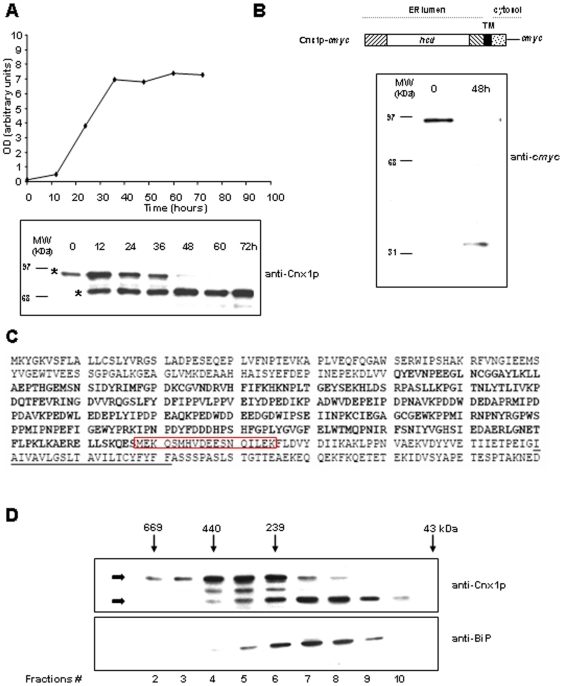
Calnexin is cleaved in the lumenal domain under normal conditions. (A) Time-course cleavage of calnexin. Samples corresponding to 10 µg of protein extracts from cells expressing endogenous calnexin at different time points of growth were loaded on a 10% (wt/vol) SDS-PAGE gel. Calnexin was detected by immunoblotting with an anti-Cnx1p rabbit polyclonal serum (at a 1∶30,000 dilution). The positions of the molecular mass markers (in kDa) are indicated on the left. The two forms of calnexin detected are identified with an asterisk (*). Concomitantly, the OD_595_ of cells expressing endogenous calnexin was measured at each time point and graphed in correlation with the cleavage of calnexin as assessed by Western blotting with anti-Cnx1p rabbit polyclonal serum. (B) Anti-*cmyc* immunoblot detection of *c-myc*-tagged calnexin before and after cleavage. Samples corresponding to 10 µg of protein extracts from cells expressing a *cmyc*-tagged version of calnexin (Cnx1p-*cmyc*) at OD_595_ = 0.3 or OD_595_ = 7 were loaded on a 15% (wt/vol) SDS-PAGE gel. Calnexin was detected by immunoblotting with the 9E10 anti-*cmyc* mouse mAb (at a 1∶500 dilution). The positions of the molecular mass markers (in kDa) are indicated on the left. (C) Region of calnexin spanning the site of cleavage. The region of cleavage of calnexin was determined by a series of truncation mutants and tandem mass spectrometry analysis of the small *cmyc*-tagged fragment of calnexin detected in Figure 6B. In this sequence, the amino acids in bold correspond to the highly conserved central domain (hcd) and the underlined residues correspond to the transmembrane domain (TM). The region delimited in red contains the cleavage site of calnexin. (D) Gel-filtration fractionation of uncleaved (full length) and cleaved calnexin. Samples from cells expressing endogenous calnexin cultured for 24 h were subjected to gel-filtration analysis. Equal volumes of fractions 2 to 10 were loaded on an 8.5% (wt/vol) SDS-PAGE gel and immunoblotted with anti-Cnx1p rabbit polyclonal serum (at a 1∶30,000 dilution) or anti-BiP rabbit polyclonal serum (at a1∶30,000 dilution). Arrows denote the migration of molecular mass standards (thyroglobulin, 669 kDa; β-amylase, 200 kDa; bovine serum albumin, 68 kDa; carbonic anhydrase, 29 kDa). The two forms of calnexin detected are identified with black arrows.

The TM and the cytosolic tail of calnexin are important domains in at least two situations leading to apoptosis, ER stress and inositol starvation. Interestingly, in time-course experiments to measure the levels of calnexin expression by Western blotting with WT cells, we observed that calnexin undergoes cleavage in a time-dependent manner under normal culture conditions (Figure 6A). The size downshift of calnexin begins at 12 h of culture corresponding to OD_595_∼1, thus in early exponential phase (Figure 6A). Complete cleavage of calnexin of calnexin is observed after 48 h of culture when cells reach an OD_595_ of 7. It is important to note that this downshift in size is observed in medium containing inositol, and that the same pattern is observed whether the calnexin gene is in the genome or calnexin is expressed by a plasmid in a *Δcnx1* background. The same downshift in the size of calnexin was observed in media without inositol (not shown). To determine whether the cleavage occurs closer to the N- or C-terminal end, we used a version of calnexin *cmyc*-tagged at its C-terminus. At time-point 0 corresponding to OD_595_ 0.3, we detected the full-length calnexin by Western blotting against *cmyc*. However, after 48 h of culture, corresponding to OD_595_ 7, we detected by anti-*cmyc* immunoblotting a small calnexin fragment migrating at *Mr* of 31 kDa, which apparently is not cleaved further. This indicates that the cleavage takes place at a site approximately within C-terminal third of the protein (Figure 6B). The band observed near 31 kDa migrated more slowly than the C-termTM_Cnx1p_cmyc construct [72], indicating that calnexin is probably cleaved within its lumenal domain. An approximation of the cleavage site was obtained by analysis of a collection of different calnexin mutants created in the laboratory (not shown), and by MS/MS analysis of the *cmyc*-tagged small fragment of calnexin isolated by immunoprecipitation from cell extracts after 48 h of growth. This confirmed that calnexin is cleaved in its lumenal domain near the TM domain within a sequence of residues that is framed in red in Figure 6C. To further investigate the cleavage of calnexin, protein extracts from cells cultured for 24 h were fractionated by gel-filtration. Full-length calnexin and the cleaved-version of calnexin did not elute in the same fractions, indicating that they do not associate in the same protein complexes (Figure 6D). Supporting this point, the BiP chaperone eluted in the same fractions with cleaved calnexin, but did not co-elute with full-length calnexin. Collectively, these results demonstrate that under normal growth conditions calnexin is cleaved at the end of its lumenal domain giving rise to two stable moieties, probably each having a different cellular role.

## Discussion

The evidence accumulated in the past ten years demonstrate the existence of apoptotic pathways in yeasts [19], [21], [78], [79], [80], [81], [82], [83], [84], [85], [86], [87], [88]. Although numerous homologues of mammalian apoptosis factors are found in yeast, the key players of specific apoptotic processes are for the most part unknown [21]. And like in mammalian [89], [90], [91], in yeasts the interactions between cells factors within the apoptotic mechanisms are not completely described. The use of yeast models should assist in charting the core interactions of apoptotic pathways conserved in higher eukaryotes.

Recent reports point to a role of calnexin in apoptosis triggered by ER stress in mammalian cells. Calnexin was reported to act as a scaffold for the cleavage by caspase 8 of the apoptotic protein Bap31 under conditions of tunicamycin stress [70]. These authors suggested that the action of calnexin is dependent on its localisation in a specific ER sub-compartment and on its expression level [92]. These studies raised the possibility that calnexin plays a role in the early steps in the transduction of an apoptotic signal initiated by ER stress. In fission yeast, our recent results demonstrate that calnexin is involved in ER-stress induced apoptosis elicited by tunicamycin [72]. We showed that the anchoring of calnexin into the ER membrane is crucial for its apoptotic action under ER stress. The levels of calnexin increase during ER stress triggered by tunicamycin [57]. Consistently, overexpression of calnexin also induces apoptotic death; an experimental intervention probably mimicking conditions of ER stress [72].

Here, we demonstrate that starvation in inositol induces apoptotic cell death in *S. pombe* as observed by an important reduction in the ability to form colonies, Phloxin B staining, metacaspase activation, DNA breakage and nuclear fragmentation. This apoptotic death is dependent on Pca1p, the only metacaspase so far identified in *S. pombe*, and is influenced by the extensively-characterized UPR transducer Ire1p.

Inositol is a precursor for numerous molecules playing central roles in membrane integrity, cell signalling and vesicular trafficking. Many organisms have the capacity to synthesize inositol from glucose-6-phosphate by the enzyme inositol-1-phosphate synthase, encoded by the *INO1* gene in *S. cerevisiae* [35], [36]. The *S. pombe* genome does not encode an Ino1p homologue, thus sensitizing this yeast to inositol deficiency because inositol is an essential precursor [44], [45]. Moreover, *S. cerevisiae* strains deleted for *INO1* die when inositol is absent in the culture media [46], [47], [48], [49], [50], [51]. Numerous screens performed in *S. cerevisiae* demonstrated that deficiencies in several pathways can lead to inositol auxotrophy, indicating that inositol biosynthesis is linked to diverse cellular processes [40], [41], [93], [94], [95], [96]. *S. cerevisiae* cells cultured in media depleted of inositol show also an activation of the UPR pathway, which shuts off when inositol is replenished in the media [42], [43], [97].

The apoptotic phenotypes observed following inositol depletion could be due to an imbalance of ER calcium, as in mammals it was shown that the IP_3_ receptor (IP3R) is a regulator of ER calcium, and calcium is pro-apoptotic in certain situations [98]. IP3R favours Ca^2+^ release from the ER after binding of its IP_3_ ligand. Although no homologue of IP3R was found in yeast to date, it was shown that IP_3_ could trigger the calcium release from vacuoles [34]. The lack of inositol in the culture medium of fission yeast could lower the IP_3_ concentration in the cytosol leading to inhibition of calcium release from ER or vacuoles. It is known that an increase in the calcium concentration in the cytosol is a condition triggering apoptosis [99]. On the other hand, some studies suggest that a high calcium concentration in the ER protects against apoptosis in particular situations [100], [101]. In this sense, an inhibition of regulated calcium release by low levels of IP_3_ could imbalance the Ca^2+^ concentration in the ER and/or the vacuoles eventually triggering apoptosis. Moreover, numerous studies also demonstrate that lowering the inositol concentration or inhibiting IP3R induce autophagy [102], [103], [104], [105]. Autophagy could be a precursor of apoptosis when the condition inducing autophagy is maintained [106]. Thus, apoptosis induced by the lack of inositol could be the endpoint of autophagy.

Low levels of both inositol and IP_3_, and defects in IP3R signalling have been associated with autophagy in mammals [103], [107], [108]. Moreover, alterations in phospholipid levels in *S. cerevisiae* correspond to the appearance of autophagy markers [109]. Inasmuch as the induction of autophagy can lead to apoptosis, a possibility is that inositol starvation triggers apoptotic cell death via an autophagic program [110], [111]. Inositol is an essential precursor for a large number of phospholipids, and inositol deficiency deregulates the levels of phospholipids in *S. pombe* [49]. Studies in fission yeast demonstrated that deregulation in the balance of lipids induces apoptotic death [112], [113], [114]. The same group showed that deregulation of lipid homeostasis could lead to cell death via several pathways dependent or not on different players such as Pca1p, Rad9p or Pkc1p. Moreover, it was shown that valproate, a short-chain fatty acid causing inositol depletion in budding yeast, induces a metacaspase-dependent apoptotic pathway with accumulation of neutral lipids in this yeast [88], [115], [116], [117]. Hence, these observations raise the possibility that inositol deficiency triggers apoptotic death in fission yeast via the loss of lipid homeostasis.

It was shown that *INO1* is under the control of Ire1p in *S. cerevisiae* and that UPR in this yeast is tightly linked to inositol biosynthesis. Therefore, we expected that *ire1^+^* could have a role in the apoptotic pathway induced by inositol starvation in *S. pombe*, although this fission yeast does not contain a homologue of *INO1* [42], [43], [97]. We observed that deletion of *ire1^+^* decreases significantly but not totally the levels of apoptotic death provoked by the absence of inositol. This observation demonstrates that in fission yeast, the consequences of inositol deficiency are partially dependent on the major UPR transducer. The fact that deletion of *IRE1* leads to inositol auxotrophy in *S. cerevisiae* and that deletion of *ire1^+^* in *S. pombe* reduces the apoptotic effect provoked by inositol starvation suggest that Ire1p could be involved not only in inositol biosynthesis but also in inositol signalling. It is possible that Ire1p acts as an inositol sensor in both yeasts, signalling for inositol biosynthesis in *S. cerevisiae* and for cell death in *S. pombe* when cells are depleted of inositol.

Absence of the TM and cytosolic tail of calnexin increases the apoptotic phenotype due the lack of inositol in the media. By co-expression of lumenal_Cnx1p with C-termTM_Cnx1p_cmyc, we observed that the cytosolic tail anchored to the ER membrane reduced to WT levels the apoptotic effect provoked by inositol deprivation. This observation demonstrates the involvement of calnexin in this apoptotic pathway and points to the importance of the cytosolic tail and TM of this ER protein. Remarkably, these results are in contrast with our previous observations regarding the involvement of calnexin in ER-stress mediated apoptotic cell death [72]. In our previous study, we demonstrated that apoptosis triggered by tunicamycin treatment is less efficient in cells expressing only lumenal_Cnx1p, a calnexin mutant not anchored to the ER membrane [72]. In the present work, apoptosis induced by inositol starvation is more efficient when only the lumenal version of calnexin is present in the cell, suggesting that calnexin is involved in at least two different apoptotic pathways: one induced by ER stress and the other by inositol starvation. Although inositol starvation and tunicamycin treatment are two inducers of apoptosis, their mechanisms of action leading to cell death are different. Tunicamycin treatment blocks N-glycosylation, which causes folding defects resulting in severe ER stress [118], [119]. In the case of inositol starvation, while the actual mechanism leading to apoptosis remains undefined, it is known that lack of inositol affects lipid homeostasis and signalling [46], [47], [48], [49], [50], [51], [103], [108]. These significant differences may underlie the two diverging effects observed with the mutant lumenal_Cnx1p in these two apoptotic situations. Another distinction between these two apoptotic situations resides in the dependence or independence on the Pca1p metacaspase. Here we demonstrated apoptosis induced by inositol starvation is dependant on the metacaspase Pca1p, whereas in the case of ER-stress apoptotic cell death is independent of Pca1p [72]. From our studies, it is clear that calnexin is implicated in apoptotic processes, and that the TM and cytosolic tail of calnexin play important regulatory roles in these death pathways. These observations suggest that the TM and cytosolic tail of calnexin form crucial interactions playing pro- or anti-death roles depending on the apoptosis pathway.

In the course of our experiments, we came to the unexpected observation that calnexin undergoes cleavage within its lumenal domain under normal growth conditions. At late exponential phase (OD_595_ 4), calnexin is cleaved into two moieties: a large one spanning most of the lumenal domain and a small one containing a few residues of the C-terminal part of the lumenal domain attached to the TM and the cytosolic tail. This cleavage is consistently 100% efficient under normal culture conditions at the same growth stage, suggesting that this is a physiological processing of calnexin that is important for its cellular roles. In gel-filtration, full-length and processed calnexin elute in different fractions; hinting that they associate with different factors and that each calnexin fragment is involved in different cellular roles. It appears then possible that the small C-terminal moiety of calnexin interacts with other proteins in a complex that negatively regulates apoptosis induced by inositol starvation.

**Figure 7:**
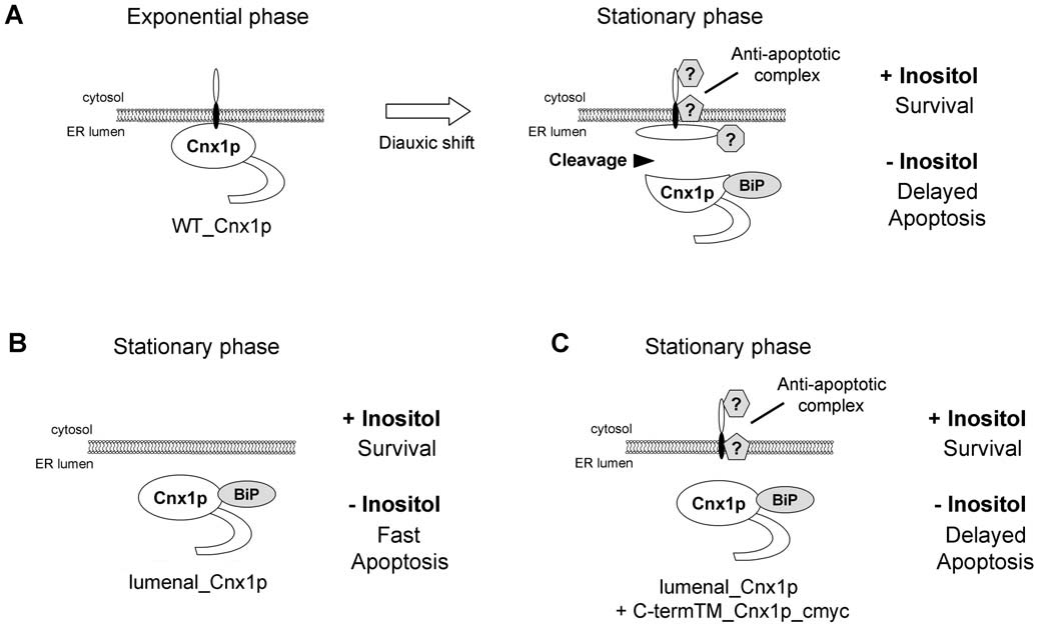
Model for the involvement of calnexin domains in the regulation of apoptosis induced by inositol starvation. (A) WT calnexin in cells growing in exponential and stationary phase. In the presence or absence of inositol, WT calnexin cells survive until they reach the early stationary phase. In early stationary phase, calnexin undergoes physiological cleavage into two moieties. The small calnexin fragment associates with cell factors into a complex that delays apoptosis under conditions of inositol starvation. (B) Cells expressing only the lumenal_Cnx1p mutant. The formation of an anti-apoptotic complex is not possible and apoptosis ensues when cells reach stationary phase. (C) Co-expression of C-termTM_Cnx1p_cmyc with lumenal_Cnx1p. The co-expression of C-termTM_Cnx1p_cmyc mimics the cleavage of calnexin when cells attain stationary phase. C-termTM_Cnx1p_cmyc mediates the assembly of the anti-apoptotic complex thus delaying apoptosis.

Based on our results obtained with the lumenal_Cnx1p and C-termTM_Cnx1p_cmyc mutants, in Figure 7 we propose a simple model explaining the role of the cleavage of calnexin in apoptosis induced by inositol starvation. Panel A depicts WT calnexin in cells growing in exponential and stationary phase. In the presence or absence of inositol, WT calnexin cells survive until they reach the early stationary phase. In early stationary phase, calnexin undergoes physiological cleavage into two moieties: a large lumenal portion, and a small one containing a few residues of the C-terminal part of the lumenal domain attached to the TM and the cytosolic tail. The small calnexin fragment associates with cell factors (that could be on either side or both sides of the ER membrane) into a complex that delays apoptosis under conditions of inositol starvation. This anti-apoptotic complex would allow autophagy to recycle inositol-containing molecules thus delaying apoptosis. However, under prolonged inositol starvation, cells finally undergo apoptotic death. In the case of cells expressing only the lumenal_Cnx1p mutant (Figure 7B), no such anti-apoptotic complex is formed and apoptosis ensues when they reach stationary phase. In panel C, co-expression of C-termTM_Cnx1p_cmyc with lumenal_Cnx1p mimics the cleavage of calnexin when cells attain stationary phase. C-termTM_Cnx1p_cmyc mediates the assembly of the anti-apoptotic complex. Interestingly, the homologue of calnexin in *S. cerevisiae* does not possess a cytosolic tail and Δ*ino1* cells, which are unable to synthesize inositol, rapidly die in the absence of this molecule [49].

Because the absence of cytosolic tail and TM reduced by 50% the apoptotic death induced by tunicamycin [72], in contrast to what we observed for inositol starvation, we propose that the cytosolic tail of calnexin and its TM have different regulatory roles in different apoptotic pathways, depending of the origin of the apoptotic signal. The apoptotic signal induced by tunicamycin treatment probably originates from the ER lumen, as this drug provokes folding defects within this organelle space. Whereas in the case of inositol starvation the apoptotic signal could originate from the cytosol or the ER membrane, as inositol is an essential precursor of molecules involved in signalling taking place in the cytosol and in the synthesis of membrane phospholipids. The difference observed for the dependency to the metacaspase in apoptosis induced by an N-glycosylation defect or an inositol depletion in *S. cerevisiae* and *S. pombe* is another indication of the differences in the two apoptotic pathways induced by these two particular conditions [72], [88], [118]. Taken together, these observations clearly indicate that calnexin is a partner in at least two apoptotic cell-death pathways, triggered by two different inducers. Further studies are needed to understand more precisely the mechanistic details of the involvement of calnexin in these apoptotic processes and to unveil whether calnexin takes part in other apoptotic pathways.

Calnexin was first identified as an ER phosphoprotein that binds calcium [120]. Soon after it was defined as glycoprotein-specific molecular chaperone binding its client proteins through lectin-glycan interactions [121], [122], [123], and later calnexin was shown to interact with non-glycosylated folding polypeptides via peptide-peptide contacts [58], [124], [125], [126]. Also from early reports, the role of calnexin in the quality control of protein folding was demonstrated [127]. Phosphorylation of the cytosolic tail of calnexin was shown to regulate its association with ribosomes and in this manner to affect translocation into the ER [128], [129]. More recently, calnexin was implicated in apoptosis induced by ER stress [70], [72], [92], and here we demonstrated its involvement in the regulation of another apoptotic pathway which is triggered by inositol deprivation. Clearly, calnexin is a multifunctional protein involved in several cellular processes. The use of the *S. pombe* model will continue to be instrumental in the elucidation of novel and unsuspected cellular roles of calnexin.

## Materials and Methods

### Yeast strains, media, and vectors

Experiments were carried out using the *S. pombe* strains described in Table 1. All strains were cultured at 30°C in Edinburgh minimal medium (here denoted as MM) supplemented with required supplements [130]. The *nmt* promoter in the vectors pREP41 and pREP42 is medium strength and expressed calnexin at levels comparable to the endogenous genomic copy of the gene [125]. pREP41 differs from pREP42 in that it contains the *S. pombe LEU2* marker instead of *ura4^+^*. The wild-type calnexin and the viable mutant *lumenal_cnx1* on pREP41 vector were used to study the effects of inositol starvation (strains SP3234-8 and SP3235-9). The unviable mutant *C-termTM_cnx1_cmyc* on pREP42 was also used to study the effects of inositol starvation, but in co-expression with the mutant *lumenal_cnx1* on pREP41 (strain SP8244).

**Table 1:** Yeast strains used for this study.

Strain	Genotype	Source
SP556	*h^+^ ade6-M216 ura4-D18 leu1-32*	Paul Nurse lab
SP3234-8	SP248 *Δcnx1::his3*+pREP41*cnx1^+^*	Elagöz et *al.* (1999) [65]
SP3235-9	SP248 *Δcnx1::his3*+pREP41*lumenal_cnx1*	Elagöz et *al.* (1999) [65]
SP8244	SP248 *Δcnx1::his3*+pREP41*lumenal_cnx1*+pREP42 *C-termTM_cnx1_cmyc*	This study
SP8167R	*h^+^ ade6-M210 ura4-D18 leu1-32 KanMX4*::*pca1* (*Δpca1*))	BIONEER
SP8212R	*h^+^ ade6-M210* (or M216) *ura4-D18 leu1-32 KanMX4*::*SPAC167.01* (*Δire1*)	BIONEER

### Starvation Assay

Cells were cultured for 43 h until saturation in MM containing the required supplements. To obtain exponentially growing cells, the cells were diluted and cultured overnight until they reached OD_595_ = 1.0. A volume of 10 ml of culture was centrifuged and the cells washed once with MM without inositol or leucine or adenine and resuspended in MM without inositol or leucine or adenine. The tests were performed at the indicated times of starvation.

### Time-course cleavage of calnexin

To study the physiological cleavage of calnexin, cells were taken at different time-points of the growth curve. Time 0 corresponds to an OD_595_ equivalent to 0.1 resulting from an original dilution from exponentially growing cells. Following time 0, a sample of cells was taken each 12 h to perform protein extraction and the OD corresponding to this time-point was measured. More cells were taken at time 0 in order to visualize proteins. Protein extracts were prepared as previously described in an immunoprecipitation buffer (50 mM HEPES pH 7, 50 mM NaCl, 1 mM CaCl_2_, 1% Nonidet P40) containing 10 mM iodoacetamide, 1 mM PMSF and 1× protease inhibitors (peptadine 1 µg/ml, leupepdine 1 µg/ml, phenathroline 1 mg/ml) [65]. Protein extracts were separated on 10% (w/v) SDS-PAGE gels. Proteins were transferred onto nitrocellulose membrane according to the manufacturer's instructions. Immunoblotting to detect Cnx1p was carried out with an anti-Cnx1p rabbit polyclonal antibody (LAR223), at a dilution of 1∶30,000.

### Quantification of luminal_Cnx1p levels

An equivalent of 10 ml of cells at OD_595_ = 1 from strains SP3235-9 and SP8244 were taken. Protein extracts were prepared as previously described in an immunoprecipitation buffer (50 mM HEPES pH 7, 50 mM NaCl, 1 mM CaCl_2_, 1% Nonidet P40) containing 10 mM iodoacetamide, 1 mM PMSF and 1× protease inhibitors (peptadine 1 µg/ml, leupepdine 1 µg/ml, phenathroline 1 mg/ml) [65]. Protein extracts were separated on 10% (w/v) SDS-PAGE gels. Proteins were transferred onto nitrocellulose membrane according to the manufacturer's instructions and colored with Ponceau red (Sigma). Immunoblotting of Cnx1p was carried out with an anti-Cnx1p rabbit polyclonal antibody (LAR223, dilution 1∶20,000) and immunoblotting of tubulin was carried out with rabbit polyclonal anti-human tubulin antibodies (Santa Cruz Biotechnologies, dilution 1∶1000). Band quantification was performed with the Quantity One software (Biorad).

### Gel-filtration chromatography

Protein extractions were performed as previously described in an immunoprecipitation buffer (50 mM HEPES pH 7, 50 mM NaCl, 1 mM CaCl_2_, 1% Nonidet P40) containing 10 mM iodoacetamide, 1 mM PMSF and 1× protease inhibitors (peptadine 1 µg/ml, leupepdine 1 µg/ml, phenathroline 1 mg/ml) [65]. A Sephacryl S-300 (Pharmacia Biotech Inc.) column (0.8 by 60 cm) was equilibrated in gel-filtration buffer (50 mM Tris-HCl, 200 mM NaCl, 10 mM MgCl_2_, 1 mM EDTA pH 7.5, 0.1% Triton X-100, 5% glycerol) at room temperature and calibrated with molecular weight standards (thyroglobulin, 669 kDa; β-amylase, 200 kDa; bovine serum albumin, 68 kDa; carbonic anhydrase, 29 kDa). The void volume was calculated as 12.5–13 ml. The sample was loaded on the column and eluted with the gel-filtration buffer. After 12.5–13 ml, 30 fractions of 1 ml were collected and an equal volume of each fraction was loaded on an 8.5% SDS-PAGE gel. Cnx1p and BiP were detected in each fraction by immunoblotting with the corresponding antibodies.

### Mass spectrometry analysis

Immunoprecipitation was performed as previously described using cells expressing a C-terminal *cmyc*-tagged version of Cnx1p cultured for 48 hours [125]. Anti-cmyc mouse mAb 9E10 (1∶100 dilution) was used to perform immunoprecipitations. Immunoprecipitates were loaded and fractionated on a 15% SDS-PAGE gel, and the gel was stained with Coomassie blue. The band corresponding to the cmyc-tagged Cnx1p fragment was cut-out of the gel and analyzed by MS/MS by the Proteomics Core facility of the Institute for Research in Immunology and Cancer (IRIC), at Université de Montréal. The fragment was subjected to tryptic digestion and analyzed by nanoliquid chromatography/tandem mass spectrometry.

### Viability Assays

The survival of cells was measured by two different techniques: 1) the ability to form colonies by serial 10-fold dilutions spotted on appropriate plates; and 2) by cytometry with the vital fluorescent dye Phloxin B. For serial dilutions spotting experiments, an equivalent of OD_595_ = 1.0 was taken from cells starved in inositol for 48 h. The cells were serially diluted (10^−1^–10^−4^), spotted on solid media and incubated for 7 days at 30°C. Viability assays with the Phloxin B fluorescent vital dye was carried out as previously described after 18 h of starvation [22].

### Calcofluor staining

Samples containing 1.4×10^7^ cells were taken after 48 h of inositol starvation. Cells were washed once in 1× PBS pH 7.4, fixed for 10 min in a solution of 3.7% formaldehyde and washed once in 1× PBS pH 7.4. The cells were resuspended in 100 µl 1× PBS pH 7.4 containing 20 µg/ml Fluorescent Brightener 28 (Sigma Inc.) for 5 min. and washed once in 1× PBS pH 7.4. Finally the cells were resuspended in 1× PBS pH 7.4 to a final concentration of 5×10^7^–1×10^8^ cells/ml. Suitable quantities of cells were applied to a poly-lysine coated coverslips, washed and let dry. The slides were mounted with a mounting media (1 mg/mL *p*-phenylenediamine, 90% glycerol). Microscopy analysis was performed using a fluorescence inverted microscope Nikon TE2000U. Images were acquired using a motion-picture camera CCD coolSnapFX M® 12 bit and treated with the UIC Metamorph® software.

### Detection of Apoptotic Markers

#### Metacaspase Activation

Culture samples were taken after 18 hours of inositol starvation. Aliquots containing 1×10^7^ cells were washed once in 1 ml of 1× PBS pH 7.4 (136 mM NaCl, 25 mM KCl, 12 mM NaHPO_4_, 18 mM KH_2_PO_4_) and resuspended in 150 µl of 1× PBS pH 7.4 containing 10 µM FITC-VAD-fmk (CasPACE, Promega, Madison, WI, USA). After incubation for 20 min at 30°C, cells were washed once in 1× PBS pH 7.4 and resuspended in 100 µl 1× PBS pH 7.4 to be analysed by flow cytometry.

#### DAPI Staining

For DAPI (4′,6-diamidino-2-phenylindole) staining, samples containing 1.4×10^7^ cells were taken after 48 h of inositol starvation. Cells were fixed for 10 min in a solution of 3.7% formaldehyde, washed once in 1× PBS pH 7.4 containing 1% Nonidet P-40, and twice in 1× PBS pH 7.4. The cells were resuspended in 100 µl 1× PBS pH 7.4 to a final concentration of 5×10^7^–1×10^8^ cells/ml. Suitable quantities of cells were applied to a poly-lysine coated coverslips, washed and let dry. The slides were mounted with a DAPI-containing mounting media (1 µg/mL DAPI, 1 mg/mL *p*-phenylenediamine, 90% glycerol). Microscopy analysis was performed using a fluorescence inverted microscope Nikon TE2000U. Images were acquired using a motion-picture camera CCD coolSnapFX M® 12 bit and treated with the UIC Metamorph® software.

#### TUNEL assay

TUNEL (Terminal uridine deoxynucleotidyl transferase dUTP nick end labeling) assay was performed with the APO-BRDU TUNEL Kit (PHOENIX flow systems, San Diego, CA), essentially following the manufacturer's recommendations. Following 48 hours of inositol starvation by culturing cells in media without inositol, 1.4×10^7^ cells were taken and fixed with 1 ml of 3.7% formaldehyde. After fixation, the cell wall was digested by resuspending the cell pellet in 200 µl of sorbitol buffer (1.2 M sorbitol, 0.5 mM MgCl_2_, potassium phosphate, pH 6.8) containing 5 mg/ml of lysing enzymes (SIGMA), and incubating for 90 min at room temperature, followed by 30 minutes incubation at 37°C. The cell pellet was resuspended in 500 µl of permeabilization solution (0.1% Triton in 0.1% sodium citrate) and let on ice for 2 minutes, washed twice with 400 µl of WASH solution and incubated in 50 µl of TUNEL solution for 30 min at 30°C. After incubation, the cells were washed twice in WASH solution and incubated 30 min at room temperature in the dark with 100 µl antibodies solution (anti-BrdU antibodies). Staining of the cells was analyzed by flow cytometry (FACS).

### Flow Cytometry Analyses

Cells were stained with Phloxin B or with FITC-VAD-fmk as described above. Flow cytometry analyses were performed using a FACS Calibur (Becton Dickinson Biosciences) device, on 10,000 cells. Emission from the argon LASER was at 488 nm; emission settings were 515–545 nm (filter FL-1) for FITC-VAD-fmk or 560–600 nm (filter FL-2) for Phloxin B staining. The percentage of positive stained cells was determined as the population of fluorescent cells with a higher fluorescent intensity than a stained negative control. Parameters of the stained negative control were adjusted with an unstained negative control. Each experiment was repeated three times.

### Statistical Analysis

The significance of the variations of results among strains was determined by a Student's *t* test.

## Supporting Information

Figure S1 Starvation in adenine or leucine does not induce apoptotic cell death (A) Survival of cells cultured for 12 h and 24 h in MM with or without leucine was assayed by serial dilution on media containing leucine. Samples of 10 µl of four 10-fold serial dilutions (10^−1^–10^−4^) of cells at OD_595_ = 1 were spotted on selective MM with leucine, and incubated at 30°C for 7 days (see Materials and Methods). (B) Survival of cells cultured for 12 h and 24 h in MM with or without adenine was assayed by serial dilution on media containing adenine. Samples of 10 µl of four 10-fold serial dilutions (10^−1^–10^−4^) of cells at OD_595_ = 1 were spotted on selective MM with adenine, and incubated at 30°C for 7 days (see Materials and Methods).
(0.14 MB TIF)

Figure S2 Quantification of lumenal_Cnx1p Anti-Cnx1p immunoblot of lumenal_Cnx1p alone (SP3235-9) or co-expressed with C-termTM_Cnx1p_cmyc (SP8244). Samples corresponding to 20 µg of protein extracts at OD_595_ = 0.5 were loaded onto a 10% (wt/vol) SDS-PAGE gel. Calnexin was detected by immunoblotting with anti-Cnx1p antibodies. Anti-tubulin immnublot and Ponceau-red staining are shown as loading controls. Band quantification was performed with the Quantity One software (Biorad).
(0.42 MB TIF)
