# Supplementary figures and images for: Calnexin Regulates Apoptosis Induced by Inositol Starvation in Fission Yeast
Source: PLoS One. 2009 Jul 16;4(7):e6244. doi: 10.1371/journal.pone.0006244 (PMC2705804; doi:10.1371/journal.pone.0006244)

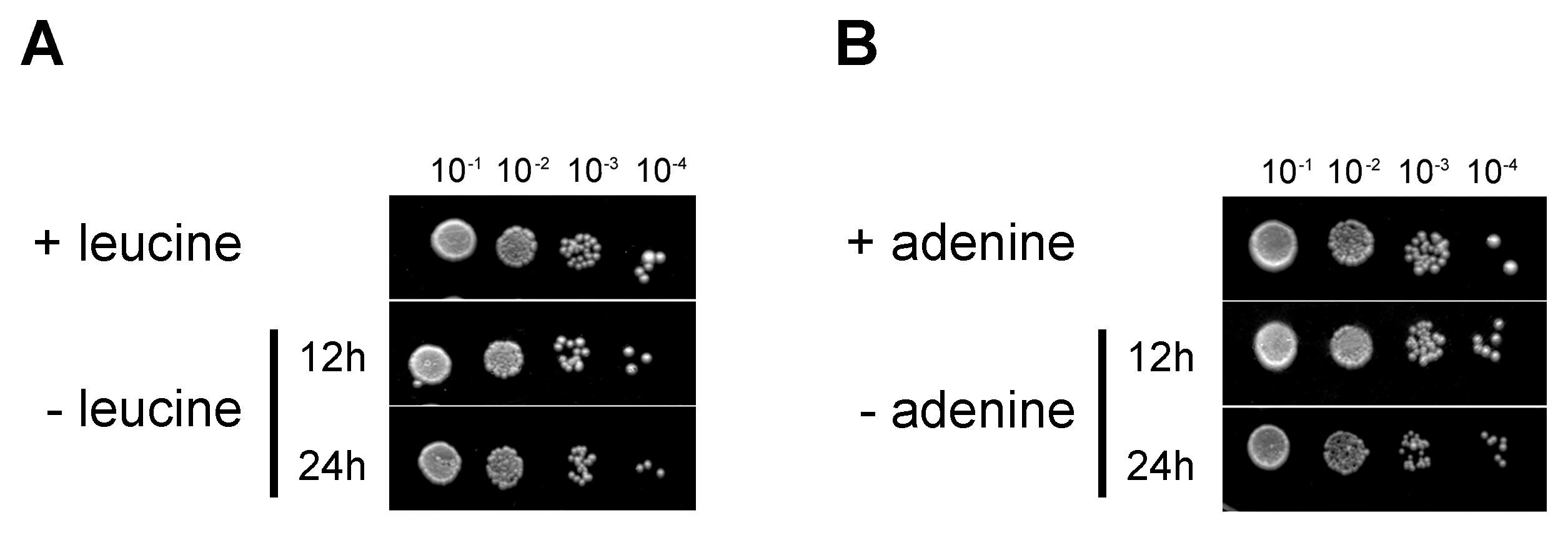

Supplement: Figure S1 — Starvation in adenine or leucine does not induce apoptotic cell death (A) Survival of cells cultured for 12 h and 24 h in MM with or without leucine was assayed by serial dilution on media containing leucine. Samples of 10 µl of four 10-fold serial dilutions (10−1–10−4) of cells at OD595 = 1 were spotted on selective MM with leucine, and incubated at 30°C for 7 days (see Materials and Methods). (B) Survival of cells cultured for 12 h and 24 h in MM with or without adenine was assayed by serial dilution on media containing adenine. Samples of 10 µl of four 10-fold serial dilutions (10−1–10−4) of cells at OD595 = 1 were spotted on selective MM with adenine, and incubated at 30°C for 7 days (see Materials and Methods). (0.14 MB TIF) [file pone.0006244.s001.tif]

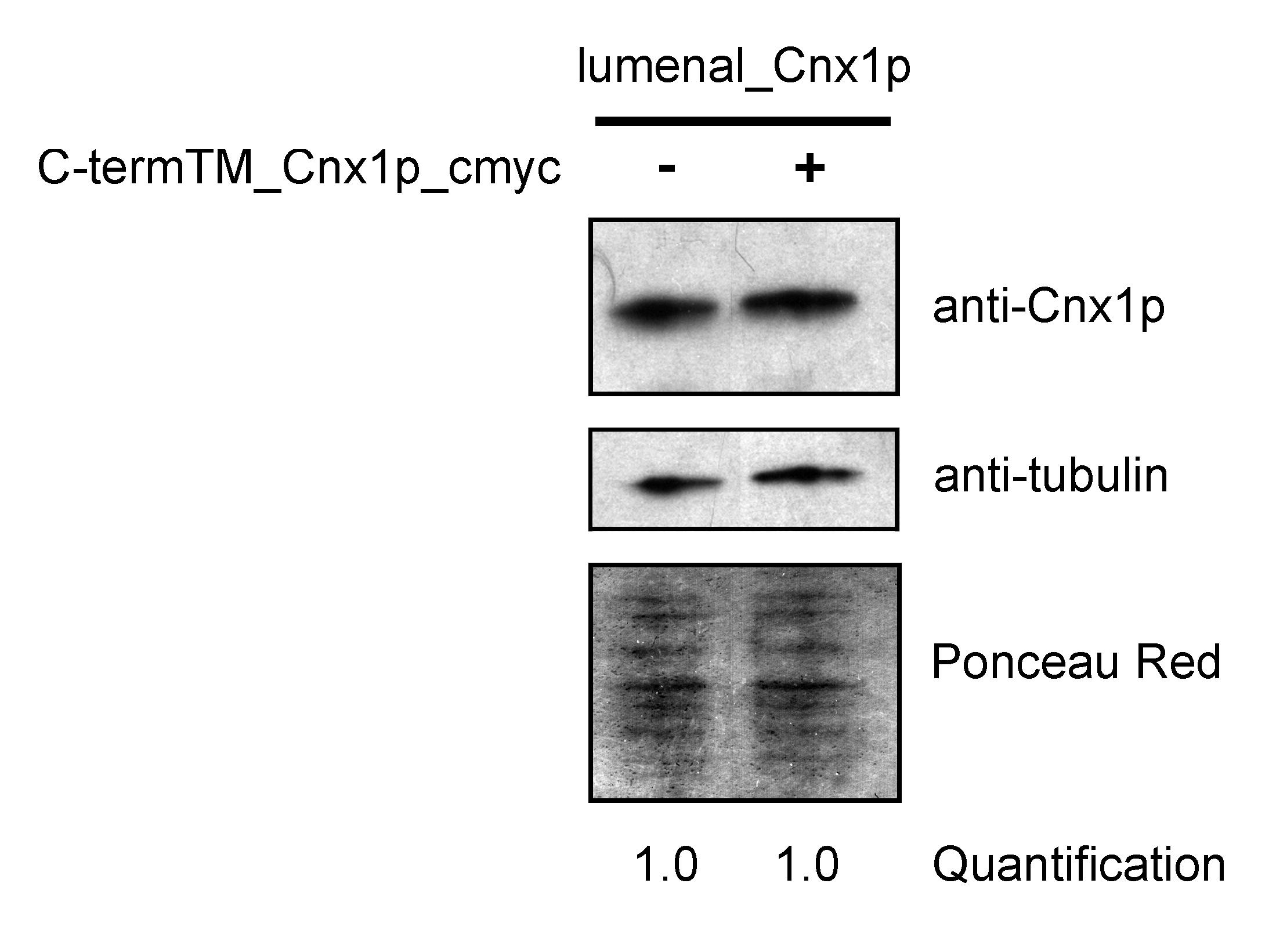

Supplement: Figure S2 — Quantification of lumenal_Cnx1p Anti-Cnx1p immunoblot of lumenal_Cnx1p alone (SP3235-9) or co-expressed with C-termTM_Cnx1p_cmyc (SP8244). Samples corresponding to 20 µg of protein extracts at OD595 = 0.5 were loaded onto a 10% (wt/vol) SDS-PAGE gel. Calnexin was detected by immunoblotting with anti-Cnx1p antibodies. Anti-tubulin immnublot and Ponceau-red staining are shown as loading controls. Band quantification was performed with the Quantity One software (Biorad). (0.42 MB TIF) [file pone.0006244.s002.tif]
